# Applied mechanics of the Puricelli osteotomy: a linear elastic analysis with the finite element method

**DOI:** 10.1186/1746-160X-3-38

**Published:** 2007-11-03

**Authors:** Edela Puricelli, Jun Sérgio Ono Fonseca, Marcel Fasolo de Paris, Hervandil Sant'Anna

**Affiliations:** 1 School of Dentistry, Federal University of Rio Grande do Sul, Porto Alegre, RS, Brazil; 2 School of Engineering, Federal University of Rio Grande do Sul, Porto Alegre, RS, Brazil

## Abstract

**Background:**

Surgical orthopedic treatment of the mandible depends on the development of techniques resulting in adequate healing processes. In a new technical and conceptual alternative recently introduced by Puricelli, osteotomy is performed in a more distal region, next to the mental foramen. The method results in an increased area of bone contact, resulting in larger sliding rates among bone segments. This work aimed to investigate the mechanical stability of the Puricelli osteotomy design.

**Methods:**

Laboratory tests complied with an Applied Mechanics protocol, in which results from the Control group (without osteotomy) were compared with those from Test I (Obwegeser-Dal Pont osteotomy) and Test II (Puricelli osteotomy) groups. Mandible edentulous prototypes were scanned using computerized tomography, and digitalized images were used to build voxel-based finite element models. A new code was developed for solving the voxel-based finite elements equations, using a reconditioned conjugate gradients iterative solver. The Magnitude of Displacement and von Mises equivalent stress fields were compared among the three groups.

**Results:**

In Test Group I, maximum stress was seen in the region of the rigid internal fixation plate, with value greater than those of Test II and Control groups. In Test Group II, maximum stress was in the same region as in Control group, but was lower. The results of this comparative study using the Finite Element Analysis suggest that Puricelli osteotomy presents better mechanical stability than the original Obwegeser-Dal Pont technique. The increased area of the proximal segment and consequent decrease of the size of lever arm applied to the mandible in the modified technique yielded lower stress values, and consequently greater stability of the bone segments.

**Conclusion:**

This work showed that Puricelli osteotomy of the mandible results in greater mechanical stability when compared to the original technique introduced by Obwegeser-Dal Pont. The increased area of the proximal segment and consequent decrease of the size of lever arm applied to the mandible in the modified technique yield lower stress values and displacements, and consequently greater stability of the bone segments.

## Background

Surgical orthopedic treatment of the mandible depends on the development of techniques allowing for larger and better adapted surfaces for bone contact, which result in faster healing processes and decreased displacement due to muscle forces [1].

The first osteotomies were performed on the mandibular body, involving a smaller area of cancellous medullary bone contact and larger muscle forces. The modification of osteotomy site to the ascending ramus of the mandible resulted in less muscle force and revealed the relationship existing between the area and type of bone tissue and healing time. Intermaxillary immobilizations, which previously took 12 weeks, were reduced to up to 5 weeks [1].

In the miniplate system introduced by Champy, only one fixation on the external cortical surface is needed, in subapical position, neutralizing traction forces in fractured mandibles [2-6]. Puricelli [1] established the use of this internal rigid fixation in orthognathic surgery, reducing intermaxillary immobilization time to 14 days. In a new technical and conceptual alternative recently introduced by Puricelli [7], osteotomy is performed in a more distal region, next to the mental foramen. The method results in an increased area of bone contact, resulting in larger sliding rates among bone segments. Conceptually, it interferes with the resistance arm of the mandible, seen as an interpotent lever of the third gender.

Currently, many of the models investigated by engineers and researchers in the area of solids mechanics are approached with the finite element analysis method. Structures involved in these models are not generally amenable to direct analytical approach, so that numerical methods must be employed for their study. This does not represent an additional problem, since numerical methods are well known, well developed and are amply employed.

The finite element analysis method has been used in the last decades for study of biological structures such as bone. In these cases, geometry of the structures is complex and irregular, and some degree of variability is observed among individuals from the same species [8]. Techniques usually employed in their analysis are too simplified and not satisfactory, often leading to incorrect results which do not adequately reflect the experimental situation.

Many studies report experimental results comparing different types of bone fixation [1-4,6,7,9]. Experiments comparing different osteotomy techniques for use in orthognathic surgery are however limited. The present work aims at comparing sagittal split osteotomy of the mandible as proposed by Obwegeser and Dal Pont and the modification introduced to the method by Puricelli, with the use of mandible models.

## Methods

**Figure 1:**
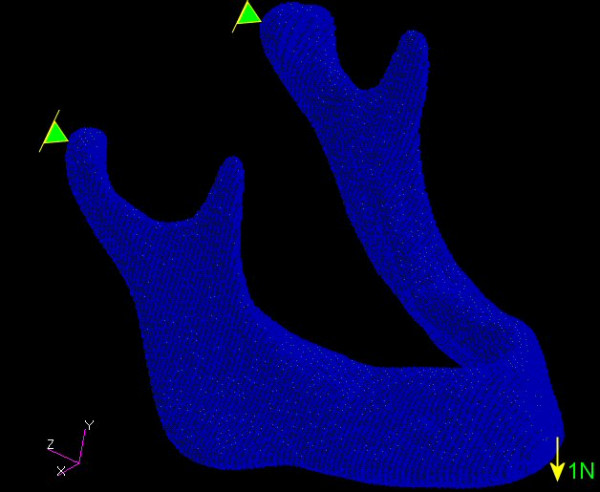
(A) Positioning of Obwegeser-Dal Pont osteotomy – Test Group I. (B) Positioning of Puricelli osteotomy – Test Group II.

Two different sagittal osteotomies of mandible were simulated *in vitro*. Three polyuretane models of mandible were selected and analyzed. The model in Test Group I was cut with a carburundum disk, as in the original Obwegeser-Dal Pont technique for sagittal osteotomy of the mandibular ramus. One of the splits was perpendicular to the mandibular ramus long axis, 13 mm from the mandibular incisure, another was parallel to the external oblique line, 5 mm lingual to it, and the third cut was 23 mm proximal to the distal border of the mental foramen, completing the osteotomy simulation. A second mandible model, Test Group II, was prepared according to the Puricelli [7] technique for sagittal osteotomy of the mandibular ramus. The procedure is similar to the described above, but is modified by an anterior extension, so that this split was 20 mm more anterior than the Obwegeser-Dal Pont split. Both procedures were bilaterally performed, so that three segments resulted from each model. The segments were then fixed to each other with monocortical four-hole Champy miniplates without space and four 5-mm stainless steel screws on each side (Figure 1). The role of fixing elements, miniplates and screws was not taken into consideration in this phase of the study. The third mandible model, Control Group, was not submitted to any treatment. Tension distribution was compared among the three groups.

The following properties of materials were considered in all analyses:

a) Polymeric resin: isotropic

• Elasticity module (E): 2,26 GPa (value established with a mechanical assay, validated by a finite element numerical model);

• Poisson coefficient (ν): 0,4 (estimated from data reported in other studies for this class of materials).

b) Steel: isotropic

• Elasticity module (E): 210 GPa;

• Poisson coefficient (ν): 0,3.

**Figure 2:**
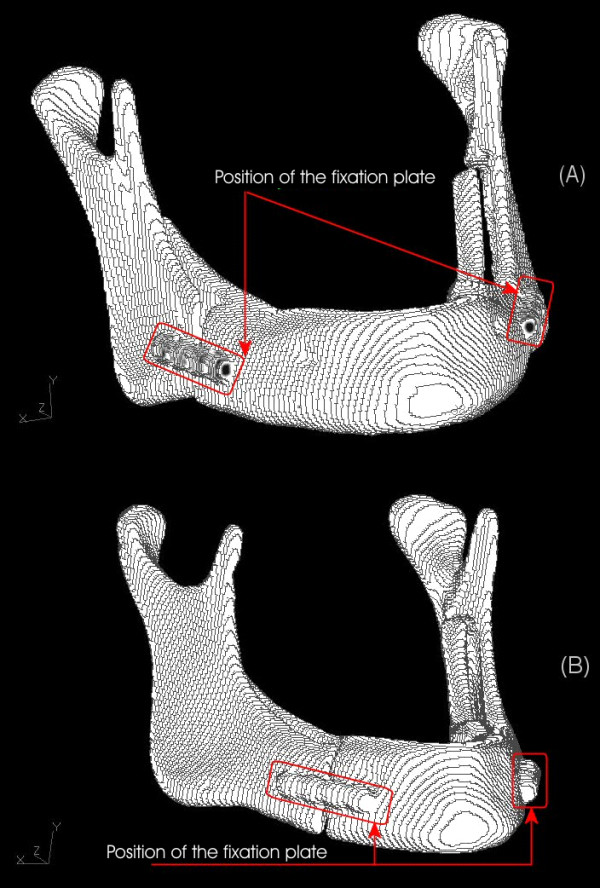
Representation of the border conditions applied (common to all models).

Samples were compared through linear elastic analysis of the voxel-based meshes generated from images obtained by computerized tomography. Tridimensional models of hexahedral finite element with enriched displacement fields were generated. At this point of the study, the use of realistic border conditions was not a concern. For comparison, mandibles were considered as balanced beams fixed in one of the endings by the condylar processes, with an unitary stress of 1 N distributed among the knots of the other ending, in the mental region (Figure 2).

In view of the large number (270,000 to 305,000) of finite elements in the meshes generated, a specific software was developed for solving equilibrium equations. Results (modal displacement and tensions) are translated into a commercial finite element software package, in which post-processing is performed.

The results compare displacement magnitude and distribution of von Mises stress. At this point of the experiment, any possible effect of contact tensions on the interface between the plate and the mandible was disregarded.

The problem is solved through the following sequence, with the use of methods: pre-processing, solution and post-processing. Three different regions were identified – material I, material II and void – according to the density of materials involved. Due to its high density, the fixation metal was easily distinguished from the polymeric resin composing the mandible model. For the binarization procedure (material/void), the DICON images generated by tomography were initially converted into BITMAPS with a 256 gray scale. The intensity value of each pixel was compared to the pre-defined threshold. If the value is below the threshold, its intensity is changed to the value below, and vice-versa. A similar procedure allows the segmentation of two materials, in which case there are two thresholds.

In this work, the pre-conditioning matrix is replaced by a vector composed of the elements from diagonal A, the rigidity matrix, and is as such known as Jacobi acceleration [10].

When the stop criterion is detected, the problem has converged and the solution (displacements) is registered in a data file. Deformations and tensions are also computed.

Results are examined with a commercial finite elements software.

## Results

**Figure 3:**
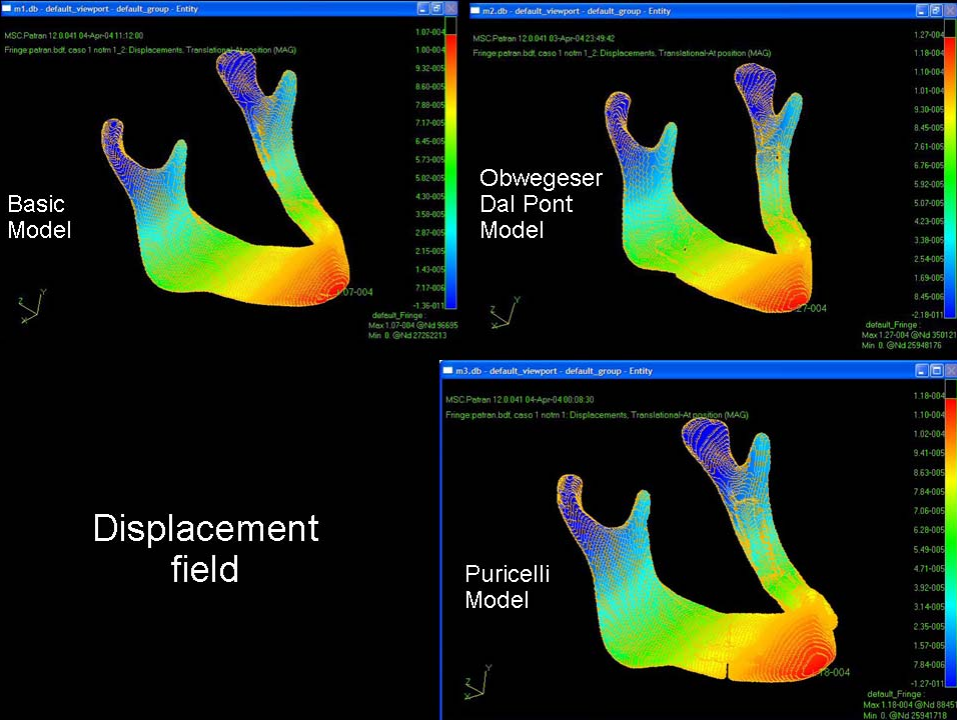
Displacement fied in the mandible, in Control Group and in the Obwegeser-Dal Pont or Puricelli models.

In Test Group I, maximum stress (von Mises tension field) was observed in the region of the rigid fixation plate. In the Test Group II model, maximum stress was smaller than in both other groups and presented a location similar to that of Control Group, in the anterior condylar neck regions (Figure 3).

**Figure 4:**
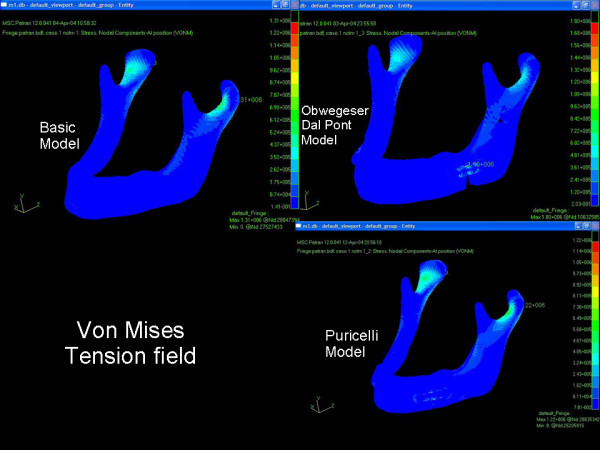
Mandible tension field, in Control Group and in the Obwegeser-Dal Pont or Puricelli models.

Test Group I presented displacement fields with values higher than those of Test Group II and Control (Figure 4).

The results are summarized in Table 1.

**Table 1:** Maximum displacement values and stress observed in the three models analyzed.

Mandible model	Maximum displacement value (mm)	Maximum von Mises stress (MPa)
No split	0.107	1.31
Obwegeser-Dal Pont split	0.127	1.80
Puricelli split	0.118	1.22

## Discussion

The finite element tridimensional mandible models used in this study were fixed to condylar processes and a unitary stress was applied to the mental region. A similar method was used by Kroon et al. [9] in an experimental *in vitro *model with polyurethane mandibles. Additional traction on the coronoid processes, however, was employed by the authors.

The cuts performed on the polyurethane models, before analyses with computerized tomography, simulated *in vivo *situations of different techniques for sagittal osteotomy of the mandible compared in this study. Fixation of segments with miniplates and screws was similarly applied.

Vollmer et al. [11] showed a good correlation between in vitro measurements and mathematical modelling. A finite element method, used in this study, can provide precise insight into the complex biomechanical behaviour of human mandibles.

The lower values of maximum tension field observed in Test Group II (1.22 MPa) as compared to Control Group (1.31 MPa) may be related to the presence and greater rigidity of rigid fixation media. The fact that these fields are equally placed in the two groups, near the anterior region of the condylar neck, shows the advantage of greater contact surface of mandibular segments resulting from the Puricelly split for stability of the fixation process.

Higher values for maximum displacement field observed in Test Group I (0.127 mm) in comparison with Test Group II and Control Group (0.118 and 0.107 mm respectively), as well as for maximum stress field (Test Group I = 1.80 MPa, Test Group II and Control Group = 1.22 and 1.31 MPa, respectively) and location on the region of rigid fixation, are probably due to a larger lever arm generated by this kind of split.

The increase in around 20 mm for the area of the proximal mandibular segment resulting from Puricelli osteotomy suggests that, *in vivo*, a larger and more adjusted medullary bone surface of contact among bone fragments and a decrease in size of lever arm are obtained. These results also suggest greater stability of bone segments and surgical results provided by the diminished lever arm. A larger surface of bone contact results in faster healing, decreased displacement due to muscle activity and, in consequence, reduced periods of intermaxillary immobilization.

The models of bone structure originated from computerized tomography result in geometrically complex structures. The mechanical analysis of these structures demands numerical methods for solving equilibrium equations. The technique used in the present work transforms each pixel (smallest 3D unit of an image) into a hexahedral finite element. Depending on the resolution in which the structure is digitalized, a mesh with hundreds of thousands of finite elements may be generated [12,10]. This results in systems of linear equations with millions of unknown elements to be discovered. The conventional finite element method employs matrix techniques for solving equilibrium equations that are limited mainly by the memory available to the computer. In other words, the work with linear equation systems of this magnitude is not possible for personal computers, and even for more refined stations. Since access to supercomputers is still restricted, an iterative method was established to solve the equilibrium equations without great computer expenses.

Despite its many advantages, the use of digitalized meshes with the EBE-PCG algorithm presents some problems. The introduction of artefacts into the images may constitute a problem. The figures show examples of serrate borders existing between two different types of material, or even between the material and its external border, in a 2D finite elements mesh composed from digital images, compared to what would be the physical limit as represented by a continuous line.

Guldberg, Hollister and Charras [13], however, showed that fluctuations in average stress invalidate each other, which means that in average stresses in the extremities are equivalent to the analytical models tested by the authors.

EBE-PG is an interactive method, meaning that for each step from an initial estimate for the variables under study, new values are generated for these same variables. The equation system, therefore, is not solved in one step only but in "n" steps, until a convergence criterion is reached allowing for a precise solution. In other words, although saving computer resources the method results in considerably increased processing time.

The thresholding step is largely dependent on the nature of the image, and thresholds are generally based in heuristic criteria. In many cases, the objective is only to separate regions with material from those without material. In this work, images generated are binarized. Simple thresholding can not be used in real biological models since, depending on the degree of resolution used, each voxel may present a different density value.

## Conclusion

The increased vestibullary bone area resulting from sagittal osteotomy, according to the Puricelli method, presents several advantages besides better visual access and transoperative manipulation. These advantages include: larger bone surface of contact with faster healing; decreased displacement due to muscle forces; and, in consequence, reduced time of intermaxillary immobilization.

Using the Finite Element Method for calculating state variables, the present work showed that Puricelli [7] osteotomy of the mandible results in greater mechanical stability when compared to the original technique introduced by Obwegeser-Dal Pont. The increased area of the proximal segment and consequent decrease of the size of lever arm applied to the mandible in the modified technique yield lower stress values and displacements, and consequently greater stability of the bone segments.

## Competing interests

The author(s) declare that they have no competing interests.
